# Functional value of elytra under various stresses in the red flour beetle, *Tribolium castaneum*

**DOI:** 10.1038/srep34813

**Published:** 2016-10-06

**Authors:** David M. Linz, Alan W. Hu, Michael I. Sitvarin, Yoshinori Tomoyasu

**Affiliations:** 1 Department of Biology, Miami University, 700E High St. Oxford, OH, 45056, USA; 2 Department of Entomology, University of Kentucky, S-225 Agricultural Science Center North, Lexington, KY, 40546, USA

## Abstract

Coleoptera (beetles) is a massively successful order of insects, distinguished by their evolutionarily modified forewings called elytra. These structures are often presumed to have been a major driving force for the successful radiation of this taxon, by providing beetles with protection against a variety of harsh environmental factors. However, few studies have directly demonstrated the functional significance of the elytra against diverse environmental challenges. Here, we sought to empirically test the function of the elytra using *Tribolium castaneum* (the red flour beetle) as a model. We tested four categories of stress on the beetles: physical damage to hindwings, predation, desiccation, and cold shock. We found that, in all categories, the presence of elytra conferred a significant advantage compared to those beetles with their elytra experimentally removed. This work provides compelling quantitative evidence supporting the importance of beetle forewings in tolerating a variety of environmental stresses, and gives insight into how the evolution of elytra have facilitated the remarkable success of beetle radiation.

Coleoptera (beetles) is one of the most successful orders on the planet, representing almost 25% of the described animal species^1^,^2^,^3^. The holometabolous development of beetles along with their diverse body shapes and sizes has likely played a role in their success, however, there is an important morphological feature that is thought to have been central to the evolutionary dominance of Coleoptera: their elytra^1^,^4^,^5^. Elytra are evolutionarily modified, beetle-unique forewings located on the second thoracic segment. These wings are highly sclerotized, and act as a hardened shield covering the dorsal surface of the beetle. Elytra also cover and protect the fully functional membranous hindwings of beetles, which are located on the third thoracic segment. The presence of elytra is thought to have allowed beetles to expand into a variety of niches by protecting the beetle against an array of harsh environmental factors, while also protecting their hindwings from damage and thus their ability to fly. Interestingly, although this statement appears quite often in literature (ranging from research articles and textbooks to books geared toward a more general audience)^1^,^3^,^4^,^6^,^7^, surprisingly few studies have quantitatively demonstrated the diverse benefits provided by elytra in tolerating multiple environmental stresses.

In regard to desiccation tolerance, elytra and the subelytral space (the space beneath the elytra) in beetles have previously been implicated in body water retention and respiration^8^,^9^,^10^. However, many of these studies were done in beetles from arid environments, with some species possessing fused elytra and/or no flight wings (see ref. 4 for fused elytra, and refs 8, 9, 10 for studies using those beetles), hence it is still elusive how more generic (un-fused) elytra play a role in water retention and if the presence of elytra is critical for desiccation tolerance in beetles. In addition, to our knowledge, the protective functions of elytra against other environmental stresses, such as predation, damage on hindwings, or cold, have not yet been empirically tested.

Here, we used the red flour beetle (*Tribolium castaneum*) as a model and quantitatively evaluated the functional significance of elytra under various stresses. *Tribolium* is an emerging model organism, offering a variety of genetic and genomic tools such as the fully sequenced and well-annotated genome^11^, established gene knock-down and knock-out techniques^12^,^13^,^14^,^15^,^16^,^17^, as well as the comprehensive online resources^18^,^19^. Using *Tribolium*, we have been investigating the molecular mechanisms underlying the evolution and diversification of beetle elytra^20^,^21^,^22^,^23^,^24^. In addition to these molecular and genetic studies, we reasoned that this beetle could also be a suitable model to test the functional significance of elytra under various environmental stresses, because of (i) ease of culturing them in the uniform flour for minimizing food related variation, (ii) the ease of collecting a large number of beetles in a short period of time for staging and sexing them, (iii) their short life cycle for a quick assay, and for minimizing developmental variation, and (iv) available isogenic lines for minimizing influence of genetic variation (see refs 25 and 26 to review the benefits of *Tribolium* as a model). In addition, *Tribolium* retain functional hindwings and the ability to fly (albeit not enthusiastic flyers), and their elytra are kept un-fused after eclosion, providing a more typical (and ancestral) context to study the function of beetle elytra.

In this study, we subjected the beetles with their elytra surgically removed (ER, Fig. 1a) to four distinct environmental challenges, and evaluated the impact on the survival of beetles lacking elytra. We first examined how elytra may protect hindwings from damage while living in the culture flour. We then examined how elytra may shield *Tribolium* from predatory attacks, by using a wolf spider (*Pardosa milvina*) as a model predator. We also examined the role of elytra in desiccation stress by placing beetles at varying levels of relative humidity (RH). Finally, we subjected beetles to a cold-shock and measured their ability to withstand rapid temperature changes. We found that, in each assay, the presence of elytra conferred a significant advantage to the beetle. Taken together, our work provides compelling quantitative evidence supporting the importance of beetle forewings in tolerating a variety of environmental stresses, and gives clues as to how the evolution of elytra has contributed to the remarkable evolutionary success of the coleopteran order. In addition, the unique desiccation and predation assay systems established in this study will be useful to further decipher physiological, behavioral, and molecular mechanisms underlying how insects deal with these challenges.

## Results

### Elytra are essential for protecting the flight wings from damage in *Tribolium*

**Figure 1:**
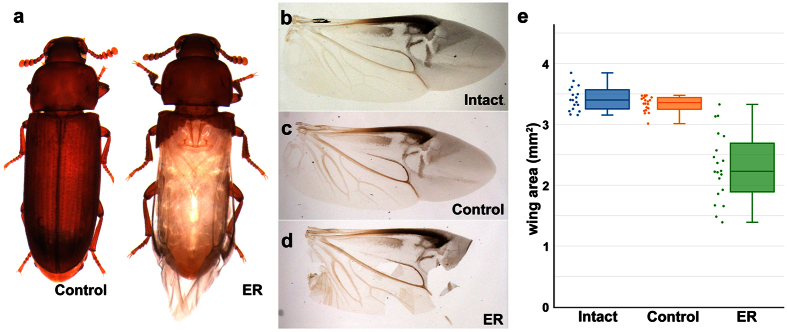
Wing damage assay. (**a**) Control and elytra surgically removed (ER) beetles. (**b–d**) Intact hindwing from a one-week-old adult (**b**), as well as control (**c**) and ER (**d**) after 28 days of the assay. (**e**) Boxplot showing intact hindwings (n = 18), as well as control and ER hindwing area (n = 20 each) after 28 days of movement in flour. The ER hindwings are significantly smaller than the control hindwings (Nonparametric Wilcoxon test, chi square = 36.0093, p < 0.0001).

It is often assumed that elytra protect the more fragile membranous hindwings when beetles move through environments that demand intensive physical contact^1^,^4^,^6^, which is believed to have allowed beetles to explore a variety of niches (such as under tree bark and inside the soil) without losing their ability to fly. However, to our knowledge, this role has never been empirically tested. We assessed how much damage the hindwings sustain when elytra are missing in *Tribolium*. The hindwings from one-week-old adults show no damage (Fig. 1b,e). The hindwings stayed undamaged in the control beetles even after four weeks of living inside the culture flour (Fig. 1c,e, Supplementary Fig. S1). In contrast, the size of hindwings of ER beetles was significantly reduced (chi square = 36.0093, P < 0.0001) due to severe damages sustained in the membranous region (Fig. 1d,e, Supplementary Fig. S1). These data indicate that elytra are essential in protecting the flight wings for *Tribolium* living in flour.

### *Tribolium* elytra provide significant protection against predation

**Figure 2:**
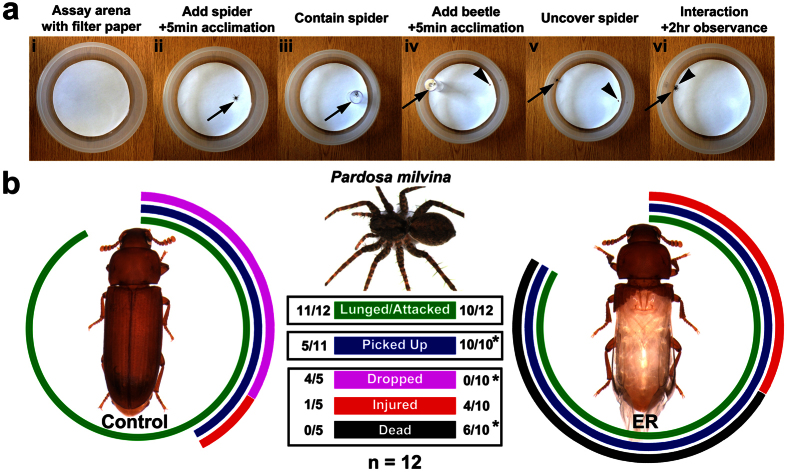
Predation assay. (**a**) Predation assay overview with spider (arrow) and beetle (arrowhead). (**b**) Outcomes of interactions between a predator and control or ER beetles. The colored arcs around the control and ER beetles are graphical representations of the outcome of the predation assay, with the length of arcs corresponding to the numbers in the boxes. Only beetles that were attacked could be picked up, and only beetles that were picked up could be dropped, injured, or dead. n = 12. Fisher’s exact test, *p < 0.05.

As a nutritious meal, insects are prime targets for predation. Some insects possess poisons, noxious chemicals, warning colors, or weaponry as their defense mechanisms^27^,^28^. The heavily sclerotized exoskeleton of beetles can also serve as an efficient protective layer against predation^4^. To examine whether the elytron, a highly exoskeletalized wing^21^, provides an additional advantage to beetles from predation, we evaluated how the absence of elytra would influence the survival of *Tribolium* during predation. We used the wolf spider, *Pardosa milvina*, as a model predator (Fig. 2), and monitored the behavior of the predator (spider) and the prey (beetle) in the predation arena (Fig. 2a. Also see Material and Methods for the predation assay procedures).

We first wondered if the removal of elytra would influence the movement of the beetle, as an alteration of prey movement could significantly influence the behavior of the predator, and thus hinder our predation assay^29^. After the initial 5 minutes of acclimation, the movement of the beetle in the predation arena (without the predator) was recorded and analyzed. Different variables of movement, such as mobility, meander, velocity, and total distance, were recorded (Supplementary Table S1). Principal component analysis (PCA) (see Material and Methods for details about PCA) returned two major principal components (PC1 and PC2). PC1 captured 66.5% of the variation in the movement of the beetle and PC2 captured 21.9%. PC1 primarily distinguished movement from non-movement, and PC2 reflected periods of high activity (Supplementary Table S2). The presence/absence of elytra had little to no effect on PC1 (Z = −0.07, p = 0.95) or PC2 (Z = −0.14, p = 0.86). Removal of elytra, therefore, does not impact their activity or motion, and hence elytron removal should not influence the initial preference of the spider.

Next, we performed the predation assay (Fig. 2a) and evaluated if the removal of elytra significantly increases the chance of predation. In the assay, we found that *Pardosa* attack control and ER beetles at the same frequency (P = 1.0000) (Fig. 2b green bar, also see Supplementary Movie S1 and S2). This further supports the idea that elytron removal has little effect on the initial preference of the spider. Following attack, however, ER beetles were more frequently picked up compared to control beetles (P = 0.0124) (Fig. 2b blue bar). Control beetles that were picked up were also more frequently dropped by *Pardosa* compared to their ER counterparts (P = 0.0037) (Fig. 2b purple bar), while picked up ER beetles were more frequently retained and killed compared to control (P = 0.0440) (Fig. 2b black bar, also see Supplementary Movie S1 and S2). Frequency of injury upon being picked up was not different between control and ER beetles (P = 0.6004) (Fig. 2b red bar).

Taken together, these data indicate that elytra do provide advantages to *Tribolium* at multiple levels during predation. First, the predatory response can be dissuaded by elytra, since control beetles were less likely to be picked up by the spider, and often dropped immediately after being picked up. Second, for more determined attacks, the act of killing (and likely feeding) on intact beetles appears to be significantly more difficult, as only ER beetles were killed in our experiments.

### Elytra provide better tolerance to desiccation in *Tribolium*

Terrestrial insects frequently encounter drying environments^30^. This desiccation stress can result from arid environments, seasonal changes in humidity, or may be induced by the substrate of their respective niche (i.e. dry soil, sand, or grain). Previous work has shown that water can be lost directly through the integument of insects, and also through the segmental spiracles during respiration (see ref. 31 for review). The elytra in beetles cover the dorsal integument as well as the abdominal spiracles, and have been shown to reduce water loss through these tissues by providing the subelytral space^8^,^9^,^10^,^32^,^33^,^34^. We hypothesized that elytra help *Tribolium* tolerate desiccation stress, allowing them to survive better in the dry flour-based environment.

To test this hypothesis, we have established a *Tribolium* desiccation assay system (Supplementary Fig. S2. Also see Materials and Methods). In addition to the presence and absence of elytra, there are at least two additional factors that could significantly influence the desiccation tolerance; sex and food consumption. Many insects (including *Tribolium*) have the ability to produce water through metabolic processes^27^,^35^, thus food consumption could help beetles survive better in a dry condition. Sex can also influence their stress tolerance, as females of some insects are known to reallocate energy from oocytes to survive a stressful environment (for review see refs 36 and 37). Therefore, we took those factors into account, and evaluated the survival rate of beetles for each sex (virgin male and female), at three different RH (0%, 70%, and 100%), with or without food consumption (+/− food), and with or without elytra (control and ER). We tested all possible conditions, except the 100% RH + food condition due to confounding fungal growths that appeared in the flour after the first week of the assay.

**Figure 3:**
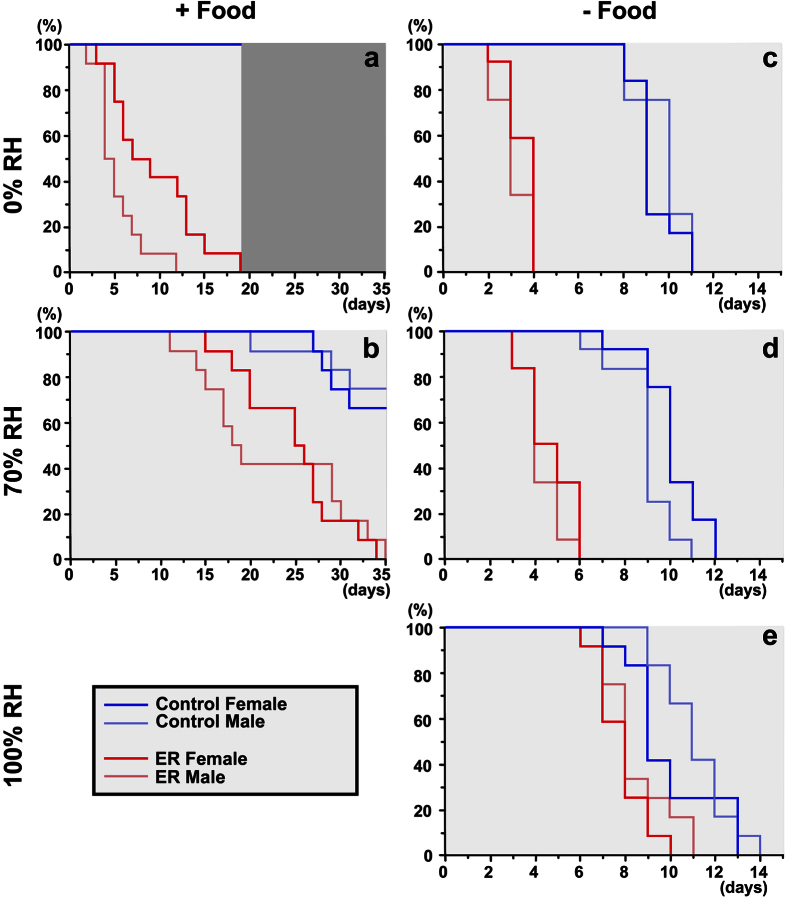
Desiccation assay. Survival curves of male (light color) and female (dark color), control (blue) and ER(red) adult *Tribolium* at various RH, with (+) and without (−) food. (**a**) 0% RH +food. (**b**) 70% RH + food. (**c**) 0% RH -food. (**d**) 70% RH -food. (**e**) 100% RH -food. In each condition, the assay was continued until all ER beetles were dead. Scale of (**a**,**b**) are 35 days. Scale of (**c**–**e**) are 15 days. n = 24.

With food provided, the control beetles survived very well even at 0% RH. 100% of the control beetles survived for the entire 19 days of assay at 0% RH (Fig. 3a). This result indicates that *Tribolium* has a very high tolerance to dry conditions, likely with the help of metabolic water produced from food consumption. Removing elytra drastically reduced their survival at 0% RH even with food provided (P < 0.0001) (Fig. 3a, Supplementary Table S3), suggesting that elytra are indeed critical for their ability to survive in a dry condition. A caveat to this interpretation is that elytron removal somehow impacted their general health and caused high mortality that is not directly related to desiccation tolerance. However, providing humidity (70% RH) increased their survival, with 50% mortality of ER beetles occurring between 24 and 25 days compared to between 5 and 6 days at 0% RH (Fig. 3b, Supplementary Table S4). Therefore, high mortality observed at 0% RH in the absence of elytra is mainly due to their inability to withstand the dry condition.

To exclude the involvement of metabolic water in desiccation tolerance, we next analyzed their survival at various RH levels without food. All control beetles died by the 11th day with 50% mortality between 8 and 9 days at 0% RH -food (Fig. 3c). The survival was only slightly improved at higher levels of RH (Fig. 3d,e) with 50% mortality occurring between 9 and 10 days in both 70% and 100% RH conditions. This result indicates that the mortality observed in the control group without food is likely due to starvation and independent of humidity. In the ER group, survival of the beetles was drastically shortened at 0% RH -food, with all ER beetles dying by 4th day and 50% mortality occurring between 2 and 3 days (P < 0.0001) (Fig. 3c, Supplementary Table S3). Interestingly, mortality of ER beetles is reduced by providing higher humidity (50% mortality between 3 and 4 days at 70% RH, Fig. 3d), almost reaching the survival of the control beetles at 100% RH (50% mortality between 7 and 8 days, Fig. 3e). Therefore, the mortality observed at 0% RH without food in the ER beetles is a result of their inability to handle the dry condition caused by the absence of elytra. Sex does not appear to affect survival of the beetles in our assay system, as we did not observe significant differences between sexes in a majority of groups tested (see Supplementary Table S5) (Fig. 3). This may be due to the fact that we used virgin beetles. Together, our desiccation assay clearly demonstrates that elytra play a critical role in tolerating dry environments in *Tribolium*, both with and without food.

### Elytra help *Tribolium* withstand a rapid cold shock

**Figure 4:**
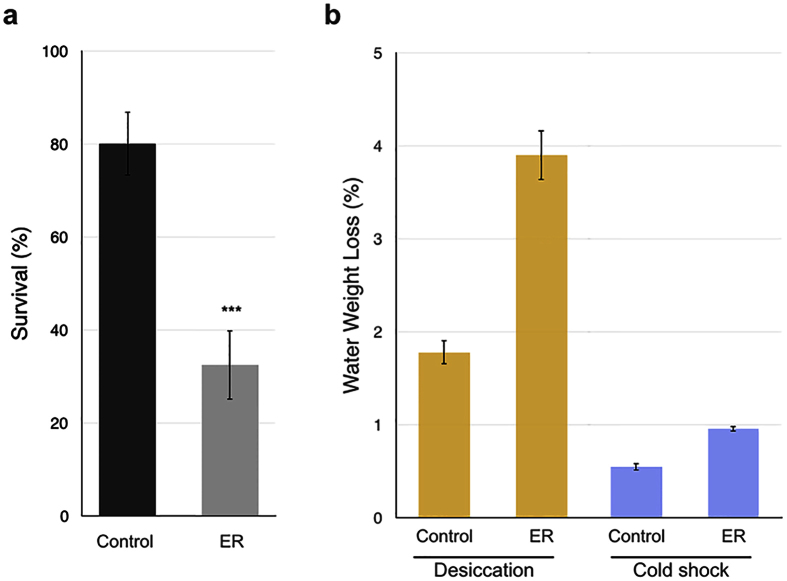
Cold shock assay. (**a**) Percent survival of control and ER beetles after a 24 hour cold shock at −4 °C. n = 40. Fisher’s exact test, ***p < 0.001. (**b**) Percent water weight lost in control and ER beetles after a 24 hour cold shock at −4 °C (cold condition) or a 24 hour desiccation period at 0% RH and 30 °C (desiccation condition). n = 40.

Cold shock is a frequent stress encountered by species living in a temperate climate. This stress can be encountered both day-to-day as well as seasonally (see refs 38 and 39 for review). To determine if elytra help protect beetles from day-to-day cold shocks, we tested the ability of *Tribolium* to withstand a short period of low temperature when elytra are removed. We performed a 24-hour cold shock at −4 °C and found a marked increase in mortality of the ER beetles compared to the control (P < 0.0001) (Fig. 4a). This result suggests that elytra indeed help protect *Tribolium* from a brief cold shock, potentially by providing the subelytral space as an insulator.

A caveat to this explanation is rapid desiccation during the cold shock experiment. As we showed above, elytra are critical for beetles to withstand desiccation. Therefore, the mortality observed in the ER beetles with a 24-hour-cold shock could be caused at least in part by desiccation. In order to assess this possibility, we measured the water loss (represented by weight loss) induced by an exposure to −4 °C for 24 hours, and compared it to the water loss induced by exposing beetles to the 0% RH environment. The exposure to the 0% RH environment at 30 °C for 24 hours does not cause any mortality even without elytra (Fig. 3c). The ER beetles lost approximately twice as much water weight as control beetles both at −4 °C for 24 hours and 0% RH at 30 °C for 24 hours, confirming an increased rate of water loss in beetles without elytra (Fig. 4b). When the two conditions are compared, the beetles exposed to the dry condition lost approximately four times the amount of water compared to the beetles in the cold condition (Fig. 4b). Therefore, a 24-hour cold shock at −4 °C did not cause a greater loss in water weight compared to beetles that underwent a 24-hour desiccation exposure at 0% RH, even though the −4 °C exposure caused mortality while the 0% RH exposure did not. These data indicate that desiccation alone cannot explain the high mortality observed in the ER beetles that were exposed to −4 °C for 24 hours, supporting the idea that elytra provide an advantage to beetles undergoing a brief cold shock.

## Discussion

### Elytra are critical in tolerating a variety of environmental stresses in *Tribolium*

It is relatively intuitive to imagine the hardened tight-fitting elytra as a dorsal shield of beetles. A multitude of literature, including research papers and textbooks, often assume that elytra protect beetles from various environmental challenges^1^,^3^,^4^,^6^,^7^. However, studies experimentally testing the function of elytra as a protective shield are surprisingly scarce. In this study, we demonstrated that elytra are indeed crucial in protecting beetles from a variety of environmental stresses. Our work provides the first comprehensive overview demonstrating the functional significance of elytra, which furthers our understanding of the significance of this structure in the evolution of Coleoptera.

#### Hindwing damage

The importance of elytra in protecting the hindwings is often assumed to be the driving force for the successful radiation of Coleoptera, by allowing beetles to explore the niches where other winged insects have difficulty dwelling without damaging their flight wings. However, despite the popularity of this statement, to our knowledge, this function of elytra has never been empirically tested. In this study, we demonstrated that hindwings sustained significant damage without the protection of elytra, even though the flour in which *Tribolium* live is relatively soft. Therefore, the protection over hindwings provided by elytra should be quite significant for beetles living in more abrasive niches, such as under the bark of trees or inside the soil or sand.

#### Predation

Our predation assay revealed that elytra are also pivotal as a protective layer against predation. It is quite interesting that elytra provide an advantage at two levels; during the initial attack where the predator quickly gives up when elytra are intact, and at the feeding step where the predator exhibits difficulties killing (and likely consuming) beetles with elytra. There might be a link between the defensive function of elytra and another defense mechanism that *Tribolium* possess, a chemical defense. *Tribolium* have two pairs of ‘stink’ glands, one in the thorax and the other in the abdomen^40^, which secrete a quinone-based chemical cocktail to repel predators^41^. Since elytra are covered with numerous sensory bristles in most beetles (including *Tribolium*)^13^,^20^, it is possible that the elytra serve as a sensitive detector for predatory attacks. Removing elytra might have lowered the ability of the beetles to sense the attack, which may have caused a delay in secreting defensive chemicals in the ER beetles. This could explain the two levels of defense advantages we observed in our assay; (i) the large sensory surface of elytra allows sensitive detection of predatory attack, which is followed by a chemical defense to dampen predator’s motivation, and (ii) the exoskeletalized surface of the elytra provides mechanical protection, making it more difficult for a predator to consume the beetle. Analyzing the behavior of the spider when the defensive gland is removed from *Tribolium* (via a gene knockdown technique) will help further our understanding of interaction between the chemical defense and the protective function of elytra. It is also worth mentioning that beetles with extensive chemical defense systems (such as lycid net-winged beetles and meloid blister beetles) tend to have soft bodies and less sclerotized elytra^42^,^43^. Therefore, there might be a significant connection between the protective function of elytra (and the hardening of the entire body) and the chemical properties of beetles during coleopteran evolution.

#### Desiccation

A possible role of elytra in desiccation tolerance has previously been studied, showing that elytra (and subelytral space) have important roles in water retention, transpiration rates, and discontinuous gas exchange patterns, mainly in beetles from arid environments^8^,^9^,^10^,^44^. Our study demonstrated that elytra are essential even in a beetle living in a more temperate environment (though flour is a dry niche compared to other natural environments), and that the lack of elytra actually causes high mortality. We also showed that this high mortality was likely caused by desiccation, as providing higher humidity reduced mortality in beetles without elytra (Fig. 3). It is yet to be determined whether the lower desiccation tolerance in ER beetles is due to the exposure of the abdominal spiracles (usually hidden underneath the elytra) or the exposure of the thin thoracic and abdominal integument. In a majority of our desiccation assays, we did not detect any differences in sexes when virgin beetles were used (Supplementary Table S5 and Fig. 3). It will be interesting to see how fertilization will influence their ability to survive in a dry condition.

#### Cold shock

Insulator and the thermal buffer functions of elytra and subelytral space were initially proposed in desert beetles, in which subelytral space helps beetles withstand high temperature (ref. 45, though this idea was argued against in ref. 46). Our study showed that elytra are critical for beetles to withstand a brief cold-shock, suggesting that elytra (and subelytral space) might also be able to buffer low temperature. A caveat to our assay was the possibility of increased mortality due to rapid desiccation, as we did not control RH in our cold shock assay system. However, as shown in Fig. 4b, the water loss was far less in the cold shock condition compared to the loss at 0% RH 30 °C, even though the former condition caused more rapid mortality. Studies examining cold and desiccation stresses in other insects show that resistance to these two stresses is often acquired mutually via similar physiological responses (see ref. 47 for review). This cross-tolerance would suggest that mild desiccation could actually improve resistance to cold shock. In our assay, however, we saw that beetles lacking elytra are more susceptible to cold shock, despite the possible advantages provided by losing more water by desiccation compared to control beetles. The exact mechanism underlying this increased mortality is yet to be determined. Direct measurement of the temperature of beetles during cold shock may be insightful to assess if elytra (and subelytral space) are in fact acting as an insulator, allowing beetles to maintain their body temperature.

### Evolution of elytra and subsequent evolutionary changes in other tissues

Elytra are not simply hardened forewings. In addition to the highly sclerotized and exoskeletalized surface, the shape and size of elytra have also been evolutionarily modified, allowing the elytra to fit exactly to the dorsal surface of the abdominal segments. The vein-derived patterns of elytra are also significantly modified, from a regular parallel pattern of impressed striae and elytral veins in some, to no trace of vein-derived structures in others^4^. Furthermore, unlike typical flight wings of insects, elytra are usually covered with numerous sensory bristles (setae), both on the veins and in the intervein regions. Considering these diverse evolutionary modifications of the beetle forewing, it is reasonable to assume that many different types of environmental stress have contributed to shaping the beetle elytra. The data presented here demonstrate that the four categories of environmental stress we tested (damage to hindwings, desiccation, predation, and cold shock) can be valid stresses that have facilitated the evolution of elytra.

The sequence of evolutionary events giving rise to the beetle forewing is still largely elusive. Fossils of protocoleopteran beetles provide a hint to a transitional state during the evolution of beetle elytra. Protocoleopteran elytra had several primitive (i.e. flight-wing like) traits, such as the lack of heavy exoskeletalization in the inter-vein regions, a larger overall size, and a more typical wing vein pattern^48^,^49^. Some of these primitive traits (such as the lack of inter-vein exoskeletalization) are still retained in Archostemata^4^, a suborder of Coleoptera, suggesting that the evolutionary changes on elytra happened in a stepwise manner. We have previously shown that at least three independent co-option events have occurred to achieve the overall exoskeletalization of the *Tribolium* elytra^21^, while neofunctionalization of a wing gene (*abrupt*) has facilitated the evolution of elytron-unique shape^24^. These evo-devo findings further support a stepwise evolution of beetle elytra.

The acquisition of elytra has triggered several secondary changes to the beetle anatomy, possibly making beetles more dependent on elytra. This poses a significant caveat to our study. For example, beetle hindwings are thinner and more fragile than the flight wings of other insects and folded into a small subelytral space^4^, suggesting that these are secondary traits due to the acquisition of elytra. Because of these characteristics, beetle hindwings are likely more susceptible to damage compared to wings of other insects. Therefore, the dependency of the hindwing on elytra to maintain its integrity is likely exaggerated in our assay system using a modern beetle (*Tribolium*) as a model. Likewise, compared to the ancestral state of Coleoptera, the very thin dorsal abdominal surface typical of modern beetles has likely caused more water loss through the dorsal cuticle and less protection against predation to beetles when elytra are removed, possibly resulting in exaggerated outcomes in our predation and desiccation assay systems. Nonetheless, we believe that the advantages of elytra in *Tribolium* that we demonstrated in our assays reflect the evolutionary advantages provided by ‘proto-elytra’ (albeit less efficiently compared to the modern elytra) in the lineage leading to beetles, thus helping us further understand how the evolution of elytra has contributed to the remarkable success of beetle radiation.

### *Tribolium* as a model to study stress tolerance

With a variety of genetic and genomic tools available, *Tribolium* is becoming a valuable model system for genetic and developmental studies^25^. Here, we show that *Tribolium* can also be an excellent model to study stress responses. In addition, we have established unique desiccation and predation assay systems, which allow us to study the response of beetles (and the spider in the predation assay) to environmental challenges with various parameters precisely controlled. The data presented here will serve as a baseline to further investigate how beetles handle desiccation and predation stresses.

Combining the assay systems we developed in this study with gene knockdown techniques, such as RNA interference (RNAi), will be quite powerful for exploring the molecular basis behind the organismal stress responses. For instance, several membrane pore molecules, such as Aquaporins, have been implicated in desiccation tolerance in insects^50^, however the detailed involvement of these molecules is still unclear. We have identified 6 Aquaporin genes in the *Tribolium* genome (DL and YT unpublished data). It will be insightful to perform the desiccation assay on beetles with the Aquaporin genes knocked down. Recently, Noh *et al*., showed that the *yellow-e* gene is important for desiccation tolerance in *Tribolium*, further supporting the benefit of using RNAi in *Tribolium* to study the molecular basis of insect stress responses^51^. RNAi can also be used to ablate specific tissues, which will be useful to determine their importance in a stress response. As discussed above, the stink gland may play a significant role in the elytron-dependent defense mechanism. RNAi for a stink gland specific gene^41^ can be used to ablate the gland, allowing us to test the protective function of elytra against predation when the chemical-based defense mechanisms are absent. Although, caution must be taken for the latter type of study, as the targeted gene can have important functions in more than one tissue (*i.e*. pleiotropic). For our study, we did not use RNAi-based ablation to remove elytra, as RNAi for the previously identified wing genes (*e.g. vestigial*, *apterous*, and *abrupt*) result in abnormalities in several tissues in addition to elytra and hindwings^21^,^22^,^24^. Nevertheless, *Tribolium* offers a key opportunity to study physiological, behavioral, and molecular mechanisms on how organisms deal with various environmental challenges.

## Materials and Methods

### Beetle culturing

Beetles were cultured on whole-wheat flour (+5% yeast) at 30 °C with 70% RH (unless stated otherwise)^15^,^23^. The *pu-11 nubbin* enhancer trap line^20^,^22^,^52^ was used for all experiments.

### Beetle preparation and elytra removal

Beetles were staged and sexed at the pupal stage, and kept separately until they reached the adult stage. Only one-week-old adult virgin beetles were used for all assays. Beetles were anesthetized using CO_2_, and their elytra were removed with a pair of micro-scissors (Elytron-Removed group: ER). The elytra were carefully cut off near the hinge instead of being forcefully pulled off to minimize injury to the beetle (Fig. 1a). The removal of the elytra also did not cause any damage to hindwings (Fig. 1a). Beetles that were staged and sexed by the same procedures, but with their elytra intact, were used as the control group.

### Wing damage assay

The beetles (12 control and 12 ER, prepared as described above) were placed individually in each well of a 48 well plate (Falcon) with 0.25 g of flour. The prepared plate was then placed in a sealed chamber (Supplementary Fig. S2) at 70% RH and 30 °C. After 28 days, beetles were removed from the chamber and their hindwings were dissected. Hindwings of one-week-old adults were used as an undamaged control (intact). Dissected wings were fixed in 100% ethanol for at least 24 hours, and mounted on slides in ethanol. The wings were documented by Zeiss Discovery V12 and AxioCam MRc5. The area of the wing was determined using Zeiss AxioVision. Wings that became folded during mounting were not included. Areas (mm^2^) were compared between the groups (n = 18 for one-week-old and n = 20 for control and ER) using a nonparametric Wilcoxon test. For the damage assay, sex was initially monitored but later combined after no sex specific differences were found. The boxplot was generated by the online Plotly tool^53^.

### *Tribolium* movement assay

To assess general beetle movement, 19 control (9 female and 10 male) and 22 ER (10 male and 12 female) beetles were used. Each beetle had a 5-minute acclimation period followed by a 5-minute trial. The movement of the beetles was recorded remotely and analyzed with motion-tracking software (EthoVision XT Version 8.0, Noldus Information Technology, Wageningen, The Netherlands). We recorded the distance traveled (cm), frequency and duration (s) of time spent immobile, mobile, and highly mobile (defined as 0, 20, and 60% changes in body position between frames (10 frames per second), respectively), total meander (turn angle/distance traveled), and velocity (distance traveled/time between frames). The effect of treatment on activity was analyzed using principal components (PC) analysis to identify variation and patterns in the dataset (see ref. 54 for details about PC analysis). Among the principal components, PC1 (movement vs. non-movement) and PC2 (periods of high activity) had eigenvalues greater than one, and thus were used in analyses^54^. Each PC retained was analyzed using a Wilcoxon test.

### Predation assay

The wolf spider, *Pardosa milvina*, was cultured as previously described^55^. Only adult female spiders were used for this study. Each spider was starved for one week prior to the trials. A round plastic bin (Rubbermaid 2.4 L #4 bowl) with a diameter of 18 cm was used as a predation assay arena (Fig. 2a). Filter paper (Whatman) was taped down at the bottom of the arena to provide sufficient traction for both beetles and spiders when they move. The filter paper was replaced and the whole bin was washed with soap (Dawn) and 70% ethanol prior to each trial to remove any possible olfactory cues left from previous trials. To start each trial, first, the spider was acclimated to the arena for 5 minutes (Fig. 2a-i,ii). The spider was then isolated with a plastic vial (Genesee wide fly vial), while the beetle was introduced into the arena for an additional 5 minutes of acclimation (Fig. 2a-iii,iv). After the acclimation step, the spider and beetle were moved to opposite sides of the arena (Fig. 2a-v). The trial began when the vial was removed so the spider and beetle could interact. Upon interaction (Fig. 2a-vi, which occurred almost immediately (less than 1 minute in all trials)), multiple variables were recorded, which include (i) whether the spider lunged at the beetle (defined as an aggressive forward motion toward the prey), (ii) whether the beetle was physically picked up by the spider, and (iii) whether the beetle was dropped (and unharmed/alive), injured (with disrupted movement), or dead. Each trial was observed for an additional 2 hours. Predation behavior was also documented by Exo Labs Camera with iPad (Apple). No spiders or beetles were reused for trials (n = 12 for each treatment). A Fisher’s Exact Test, with the FDR method to control the family-wise error rate, was used to determine the difference in observed categories^56^.

### Desiccation assay

Three different humidity conditions (0%, 70%, and 100% RH at 30 °C) were used for this assay, either with or without flour (+/−food). 24 control and 24 ER beetles were used for each condition and for each sex (prepared as described above). The beetles were individually placed in a 48-well plate. A 7 mm metal fishing weight (Unique Bargains) was placed in each well to provide the beetle with a physical object to assist in righting themselves, should they flip onto their dorsal surface and become stuck (preventing them from consuming more energy while flipped). Approximately 0.25 g of flour was measured into each of the 48 wells for the +food condition. The 48-plates were placed in a sealed chamber to maintain a constant RH (Supplementary Fig. S2). For 0% RH, approximately 200 g of Drierite (W.A. Hammond Drierite Company) was placed in the bottom of the chamber. For 100% RH, a small sponge (Genesee wide fly vial plug) saturated with water was placed in the chamber. For 70% RH, the chamber was equilibrated in a humidity controlled incubator before being sealed. A humidity indicator strip (Telatemp) was also placed in the sealed chamber to confirm the RH inside the chamber. The chambers were kept in an incubator at 30 °C. The survival of the animals was monitored every 24 hours through the transparent lid without opening the chamber. Each beetle was examined for at least 1 minute and monitored for movement. Beetles that showed no movement were considered dead. Beetles that appeared to have died were monitored for at least 3 additional days to confirm death. The difference of survival times between the experimental groups was analyzed by log-rank test.

### Cold shock assay

40 control and 40 ER beetles were used for this assay. After measuring their pooled weight (4 groups of 10) (Mettler Toledo AG104), the beetles were placed individually into 1.5 mL tubes (USA scientific) and kept at −4 °C for 24 hours using a programmable temperature bath (NESLAB RTE-140). The pooled weight was taken again after the 24 hours of cold exposure. The beetles were allowed to recover for 4 hours at room temperature, and their survival rates were determined (beetles that showed any movement after the recovery period were considered alive). A Fishers Exact Test was used to determine the difference in cold shock survival. The water loss via exposure to 0% RH was assessed by using the desiccation assay system described above (Supplementary Fig. S2) with 40 control and 40 ER beetles. The beetles were pooled (4 groups of 10) and weighed before and after the 24-hour trial. For both experiments, sex was initially monitored but later combined after no sex specific differences were found.

### Statistical analyses

JMP Pro (ver. 11.2.0) was used for all statistics. P < 0.05 was considered statistically significant in all assays. Data were analyzed with nonparametric methods when deviations from normality were present.

## Additional Information

**How to cite this article**: Linz, D. M. *et al*. Functional value of elytra under various stresses in the red flour beetle, *Tribolium castaneum*. *Sci. Rep*. **6**, 34813; doi: 10.1038/srep34813 (2016).

## Supplementary Material

Supplementary Information

Supplementary Movie S1

Supplementary Movie S2
