# Supplementary material for: Functional value of elytra under various stresses in the red flour beetle, Tribolium castaneum
Source: Sci Rep. 2016 Oct 6;6:34813. doi: 10.1038/srep34813 (PMC5052563; doi:10.1038/srep34813)
Supplement: Supplementary Information [file srep34813-s1.pdf]

Functional value of elytra under various stresses in the red flour beetle, *Tribolium castaneum*

David M. Linz[1,*], Alan W. Hu[1,*], Michael I. Sitvarin[2] & Yoshinori Tomoyasu[1]
[1]Department of Biology, Miami University, 700E High St. Oxford, OH, 45056, USA. [2]Department of Entomology, University of Kentucky, S-225 Agricultural Science Center North, Lexington, KY, 40546, USA. [*]These authors contributed equally to this work.

Supplementary materials

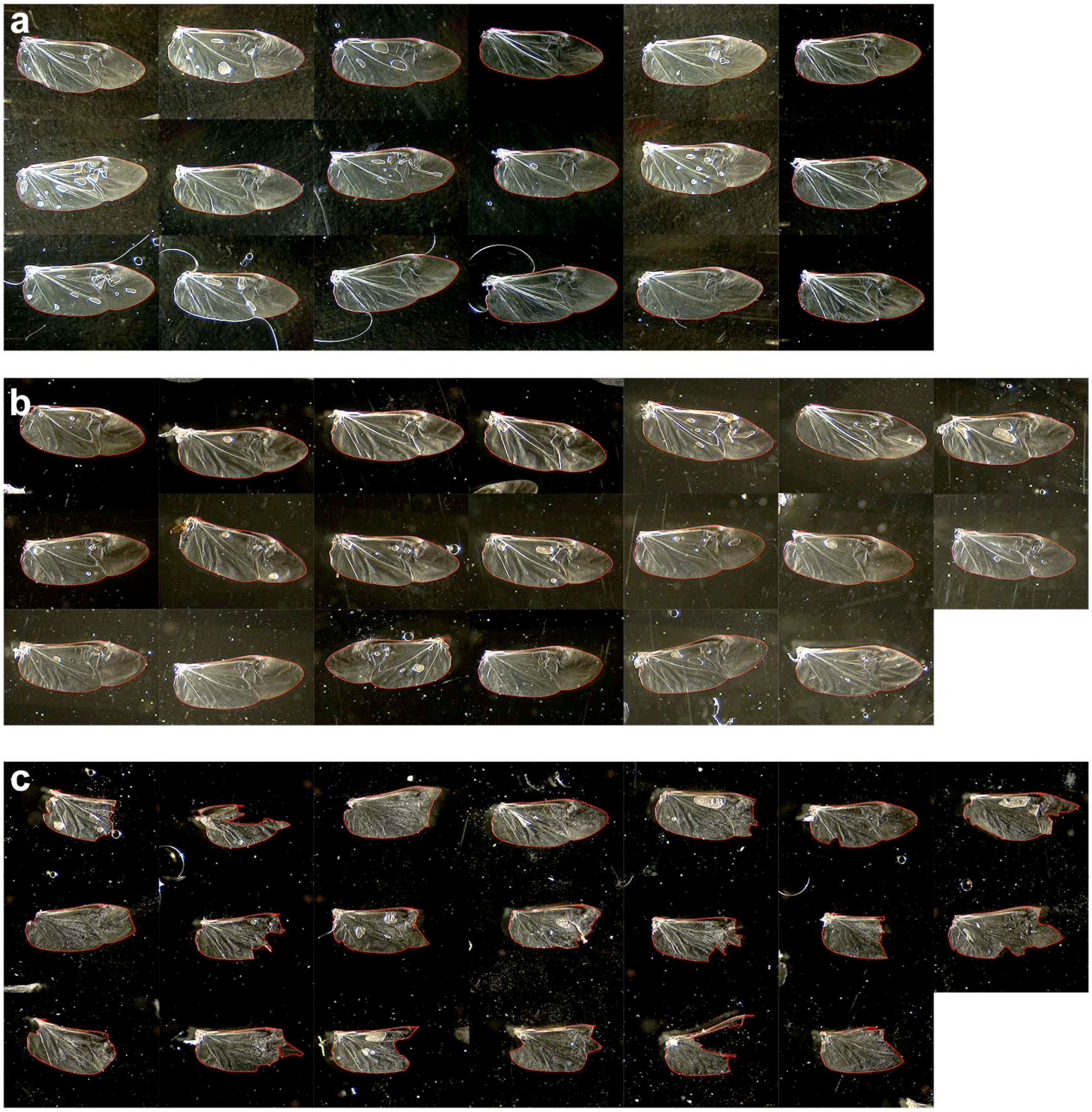

Supplementary Figure S1. Hindwings obtained by the wing damage assay. (**a**) Intact hindwings obtained from one-week-old adults. (**b-c**) control (**b**) and ER (**c**) beetles after 28 days of assay.

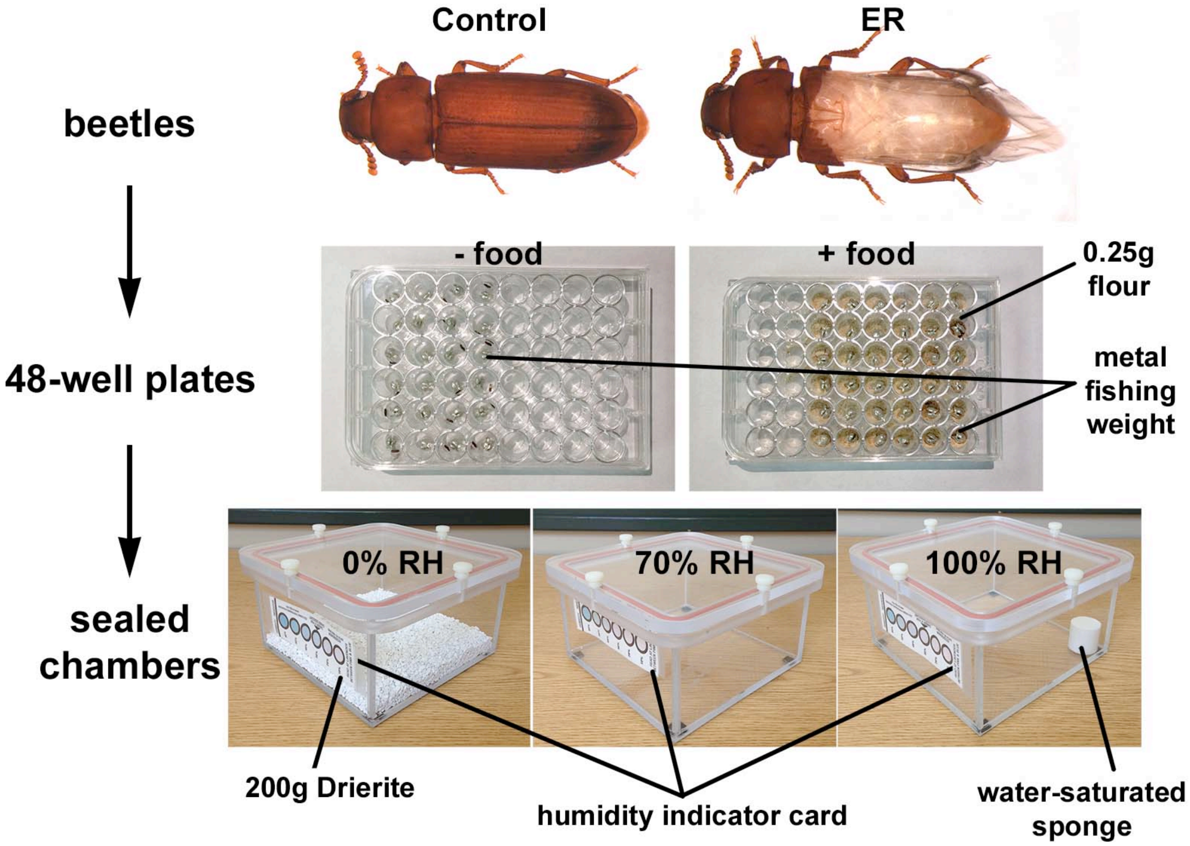

Supplementary Figure S2. Desiccation assay diagram.

Sex	CR/ER	Distance total (cm)	Meander total (deg/cm)	Immobile Duration (s)	Immobile Frequency	Mobile Duration (s)	Mobile Frequency	Highly Mobile Duration (s)	Highly Mobile Frequency	Velocity Mean (cm/s)	PC1	PC2
F	CR	287.0989476	4010747.03	42.442448	148	257.257251	152	0.3003	3	0.95699649	-1.073993072	-0.942054123
F	CR	198.7251145	3487256.363	36.936934	153	262.762765	157	0.3003	3	0.921663828	-0.768840397	-0.911772447
F	CR	301.2769775	4729653.09	55.155154	231	242.242245	258	2.6026	26	1.004256621	-0.650632599	-0.23360781
F	CR	403.1145952	1847968.723	21.02102	84	273.273277	140	5.705702	55	1.35959312	-2.776313785	0.129600201
F	CR	431.4311825	1786585.295	11.611612	61	278.278278	157	10.110109	96	1.4644912	-3.280382087	1.073060888
F	CR	202.1726314	5657756.046	74.374383	349	225.725716	350	0	0	0.673684001	0.971371514	-0.576937424
F	CR	223.4664495	3870320.716	54.454447	262	245.445452	263	0.1001	1	0.744888167	0.049873326	-0.744878968
F	CR	275.6464571	1751114.595	18.718718	119	281.181181	121	0.1001	1	0.918821517	-1.574192973	-1.066351332
F	CR	161.1663517	12464884.73	106.906901	427	190.990993	447	2.202205	21	0.539923455	2.520211275	0.131229874
F	ER	390.560918	561263.5667	3.303303	26	295.995996	34	0.7007	7	1.301869703	-3.172450178	-1.156745629
F	ER	334.926318	2646620.979	21.821825	129	276.376373	146	1.701701	17	1.134977611	-1.941272677	-0.688168625
F	ER	118.7446168	11811898.13	175.575589	537	124.42441	538	0	0	0.402941129	4.171681893	-0.08270091
F	ER	399.4203039	1743665.604	17.017016	104	277.87788	156	5.105103	51	1.361846008	-2.736107237	0.045987197
F	ER	399.4203039	1743665.604	17.017016	104	277.87788	156	5.105103	51	1.361846008	-2.736107237	0.045987197
F	ER	396.2180863	1316388.018	9.909909	51	287.287289	79	2.802801	28	1.320726949	-2.995264099	-0.606269066
F	ER	318.4604489	2567336.133	29.129125	141	270.570574	143	0.3003	3	1.061534824	-1.638227333	-0.986259585
F	ER	317.9857701	2411003.426	17.41742	110	281.881879	116	0.7007	7	1.059952575	-1.928521716	-0.960450194
F	ER	215.8654898	2542384.982	42.842841	313	257.157158	312	0	0	0.731263586	0.056063772	-0.688833459
F	ER	63.89091347	22495640.2	266.566566	280	33.433433	279	0	0	0.21673013	5.528505159	-0.419682948
F	ER	191.3592014	9737191.304	96.696698	422	201.7017	439	1.601601	16	0.648906451	1.922319977	-0.030510898
F	ER	164.6879492	12562758.67	102.702698	427	195.6957	437	1.601601	15	0.553019389	2.4413757	-0.02342787
M	CR	313.3032959	1872902.614	34.134035	186	258.457686	243	6.80678	66	1.047843866	-1.47643917	0.615353575
M	CR	301.8155504	6420009.181	28.028029	112	268.768765	138	3.203205	31	1.006051842	-1.386306298	-0.358361518
M	CR	212.1275834	8858407.642	101.401402	299	198.198196	303	0.400401	4	0.707091956	1.268392881	-0.544795094
M	CR	303.9788385	2590786.927	42.342342	210	256.456456	224	1.301301	13	1.035372874	-1.0988061	-0.599601676
M	CR	303.9788385	2590786.927	42.342342	210	256.456456	224	1.301301	13	1.035372874	-1.0988061	-0.599601676
M	CR	242.1044742	4745547.736	53.453453	285	245.445445	294	1.101101	11	0.818762237	0.019200046	-0.475805041
M	CR	168.7697062	6726323.869	137.237233	391	162.162165	392	0.600601	5	0.660920935	2.169476757	-0.29507603
M	CR	200.195084	1993841.16	43.143141	311	220.220217	480	36.636641	303	0.77337546	-0.590503101	7.01227614
M	CR	166.0373225	7461220.011	73.57357	428	226.126129	433	0.4004	4	0.553642486	1.705691019	-0.323188468
M	CR	172.3344319	7849226.164	81.581579	395	217.617617	405	0.900903	9	0.583007444	1.637152533	-0.257356708
M	ER	232.8155036	2027497.724	31.431427	158	268.568572	159	0	0	0.77605169	-0.884209841	-0.987501148
M	ER	418.1743753	471749.2636	2.402402	18	294.794795	46	2.802802	28	1.393914578	-3.467056807	-0.684158899
M	ER	303.1087782	7418963.843	59.759759	232	238.338336	250	1.901904	19	1.010362583	-0.398970817	-0.395119578
M	ER	205.4459631	5869280.131	86.086091	362	214.014008	362	0	0	0.684591447	1.163606347	-0.542565891
M	ER	139.674938	6995743.415	122.222216	738	177.877883	738	0	0	0.474609751	3.77578381	0.208671832
M	ER	114.1068667	7342309.615	187.887895	760	112.112104	759	0	0	0.380356217	4.955752453	0.332750769
M	ER	78.04600342	18025076.97	229.329318	541	70.670681	540	0	0	0.266466031	5.669396071	0.003485381
M	ER	243.8044556	8272692.653	51.951956	263	237.637634	360	10.410409	100	0.819793496	0.074918893	1.646976298
M	ER	319.2135798	6977818.536	51.351353	196	237.337331	309	11.411415	113	1.063690346	-0.904844549	1.77607449
M	ER	317.5975737	5197019.327	34.534536	148	246.946946	316	18.518517	169	1.071168035	-1.522525253	3.160329176

Supplementary Table S1. Principal Component Analysis raw data.

Measured Parameter	PC1	PC2
Distance	-0.94627	-0.0413
Meander	0.85421	0.00453
Immobility duration	0.94986	-0.00657
Immobility frequency	0.89579	0.10532
Mobility duration	-0.94058	-0.10078
Mobility frequency	0.8402	0.33066
High mobility duratior	-0.25655	0.96198
igh mobility frequenc	-0.28238	0.95505
Velocity	-0.95874	-0.01336

Supplementary Table S2. Principal Component Analysis loading matrix.

Comparison	Sex	Food/No Food	Humidity	P Value
Control vs. ER	Male	Food	0	<0.0001
Control vs. ER	Male	No Food	0	<0.0001
Control vs. ER	Male	Food	70	<0.0001
Control vs. ER	Male	No Food	70	<0.0001
Control vs. ER	Male	No Food	100	0.0007
Control vs. ER	Female	Food	0	<0.0001
Control vs. ER	Female	No Food	0	<0.0001
Control vs. ER	Female	Food	70	<0.0001
Control vs. ER	Female	No Food	70	<0.0001
Control vs. ER	Female	No Food	100	0.0038

Supplementary Table S3. Control vs. ER beetles in the desiccation assay. Survival curves analyzed by log-rank test.

0%RH +food						
Days Past	# Males Survived Control	# Males Survived ER	# Females Survived Control	# Females Survived ER	ER Total	Control Total
1	12	12	12	12	24	24
2	12	11	12	12	23	24
3	12	11	12	11	22	24
4	12	6	12	11	17	24
5	12	4	12	9	13	24
6	12	3	12	7	10	24
7	12	2	12	6	8	24
8	12	1	12	6	7	24
9	12	1	12	5	6	24
10	12	1	12	5	6	24
11	12	1	12	5	6	24
12	12	0	12	4	4	24
13	/	/	12	2	2	12
14	/	/	12	2	2	12
15	/	/	12	1	1	12
16	/	/	12	1	1	12
17	/	/	12	1	1	12
18	/	/	12	1	1	12
19	/	/	12	0	0	12

70%RH +food						
Days Past	# Males Survived Control	# Males Survived ER	# Females Survived Control	# Females Survived ER	ER Total	Control Total
1	12	12	12	12	24	24
2	12	12	12	12	24	24
3	12	12	12	12	24	24
4	12	12	12	12	24	24
5	12	12	12	12	24	24
6	12	12	12	12	24	24
7	12	12	12	12	24	24
8	12	12	12	12	24	24
9	12	12	12	12	24	24
10	12	12	12	12	24	24
11	12	11	12	12	23	24
12	12	11	12	12	23	24
13	12	11	12	12	23	24
14	12	10	12	12	22	24
15	12	9	12	11	20	24
16	12	9	12	11	20	24
17	12	7	12	11	18	24
18	12	6	12	10	16	24
19	12	5	12	10	15	24
20	11	5	12	8	13	23
21	11	5	12	8	13	23
22	11	5	12	8	13	23
23	11	5	12	8	13	23
24	11	5	12	8	13	23
25	11	5	12	6	11	23
26	11	5	12	5	10	23
27	11	5	11	3	8	22
28	11	5	10	2	7	21
29	10	3	9	2	5	19
30	10	3	9	2	5	19
31	9	2	8	2	4	17
32	9	2	8	1	3	17
33	9	1	8	1	2	17
34	9	1	8	0	1	17
35	9	0	8	0	0	17

0%RH -food						
Days Past	# Males Survived Control	# Males Survived ER	# Females Survived Control	# Females Survived ER	ER Total	Control Total
1	12	12	12	12	24	24
2	12	9	12	11	20	24
3	12	4	12	7	11	24
4	12	0	12	0	0	24
5	11	0	12	0	0	23
6	11	0	12	0	0	23
7	4	0	12	0	0	16
8	3	0	10	0	0	13
9	3	0	3	0	0	6
10	1	0	2	0	0	3
11	0	0	0	0	0	0
12	0	0	0	0	0	0
13	0	0	0	0	0	0

70%RH -food						
Days Past	# Males Survived Control	# Males Survived ER	# Females Survived Control	# Females Survived ER	ER Total	Control Total
1	12	12	12	12	24	24
2	12	12	12	12	24	24
3	12	10	12	10	20	24
4	12	4	12	6	10	24
5	12	1	12	4	5	24
6	11	0	12	0	0	23
7	10	0	11	0	0	21
8	10	0	11	0	0	21
9	3	0	9	0	0	12
10	1	0	4	0	0	5
11	0	0	2	0	0	2
12	0	0	0	0	0	0

100%RH -food						
Days Past	# Males Survived Control	# Males Survived ER	# Females Survived Control	# Females Survived ER	ER Total	Control Total
1	12	12	12	12	24	24
2	12	12	12	12	24	24
3	12	12	12	12	24	24
4	12	12	12	12	24	24
5	12	12	12	12	24	24
6	12	12	12	11	23	24
7	12	9	11	7	16	23
8	12	4	10	3	7	22
9	10	3	5	1	4	15
10	8	2	3	0	2	11
11	5	0	3	0	0	8
12	2	0	3	0	0	5
13	1	0	0	0	0	1
14	0	0	0	0	0	0

Supplementary Table S4. Desiccation assay raw data. 50% mortality in each assay is highlighted.

Comparison	CR/ER	Food/No Food	Humidity	P Value
Male vs. Female	Control	Food	0	***
Male vs. Female	Control	No Food	0	0.0611
Male vs. Female	Control	Food	70	0.6567
Male vs. Female	Control	No Food	70	0.022
Male vs. Female	Control	No Food	100	0.2579
Male vs. Female	ER	Food	0	0.0114
Male vs. Female	ER	No Food	0	0.1845
Male vs. Female	ER	Food	70	0.9473
Male vs. Female	ER	No Food	70	0.2363
Male vs. Female	ER	No Food	100	0.1808
***=100% survival - no difference between groups				

Supplementary Table S5. Male vs. female beetles in the desiccation assay. Survival curves analyzed by log-rank test.

Movie S1. Control beetle in the predation assay.

Movie S2. ER beetle in the predation assay.
